# Prognostic relevance of the neurological symptom burden in brain metastases from breast cancer

**DOI:** 10.1038/s41416-025-02967-w

**Published:** 2025-03-01

**Authors:** Ariane Steindl, Clara Zach, Luzia Berchtold, Anna Grisold, Brigitte Gatterbauer, Franziska Eckert, Zsuzsanna Bago-Horvath, Johannes A. Hainfellner, Ruth Exner, Florian Fitzal, Georg Pfeiler, Christian F. Singer, Georg Widhalm, Rupert Bartsch, Matthias Preusser, Anna S. Berghoff

**Affiliations:** 1 Division of Oncology, Department of Medicine I, Medical University of Vienna, Vienna, Austria; 2 Christian Doppler Laboratory for Personalized Immunotherapy, Division of Oncology, Department of Medicine I, Medical University of Vienna, Vienna, Austria; 3 Institute of Medical Statistics, Center for Medical Statistics, Informatics, and Intelligent Systems, Medical University of Vienna, Vienna, Austria; 4 Department of Neurology, Medical University of Vienna, Vienna, Austria; 5 Comprehensive Center for Clinical Neurosciences and Mental Health, Medical University of Vienna, Vienna, Austria; 6 Department of Neurosurgery, Medical University of Vienna, Vienna, Austria; 7 Department of Radiation Oncology, Medical University of Vienna, Vienna, Austria; 8 Department of Pathology, Medical University of Vienna, Vienna, Austria; 9 Division of Neuropathology and Neurochemistry, Department of Neurology, Medical University of Vienna, Vienna, Austria; 10 Department of Surgery, Medical University of Vienna, Vienna, Austria; 11 Department of Obstetrics and Gynecology, and Comprehensive Cancer Center, Medical University of Vienna, Vienna, Austria

**Keywords:** Breast cancer, CNS cancer, Cancer screening

## Abstract

**Background:**

Existing prognostic models for breast cancer (BC) brain metastases (BM) overlook neurological symptoms. Thus, we explored the incidence and prognostic relevance of neurological symptoms in a real-world cohort of BC patients with BM.

**Methods:**

The Vienna Brain Metastasis Registry identified BC patients with BM between 1992 and 2020, categorised by subtype: hormone receptor-positive/human epidermal growth factor receptor 2-negative (HR+/HER2−), HER2 overexpressing (HER2+), and triple-negative (TN).

**Results:**

A total of 716 patients with BM from BC were included. In total, 80% (573/716) of the patients presented with neurological symptoms at BM diagnosis. Across all BC subtypes, asymptomatic patients presented with a significantly longer median OS from diagnosis of BM compared to symptomatic patients (*p* < 0.05; log-rank test; HR+ BC 29 vs. 9 months; HER2+ BC 24 vs. 12 months; TN 12 vs. 6 months). In multivariate analysis with the BC-specific Graded Prognostic Assessment (Breast-GPA: HR:1.4; 95% CI:1.3–1.5; *p* < 0.001), the presence of neurological symptoms at diagnosis (HR:1.6; 95% CI: 1.4–1.9; *p* < 0.001) presented as independently associated with OS from time of BM diagnosis, respectively.

**Conclusions:**

Neurological burden at BM diagnosis independently predicts survival in BC patients. Our findings emphasise incorporating the symptom status in the prognostic evaluation and reassessing BM screening in high-risk patients during prospective clinical trials.

## Background

Approximately 15% of breast cancer (BC) patients will develop brain metastases (BM) during their clinical course of the disease, positioning BC as the second most prevalent malignancy—following lung cancer—causing BM among all solid tumours [1]. Despite improvements in systemic therapeutic approaches for metastatic BC resulting in enhanced control of extracranial diseases, an increasing BM incidence was reported as none of the currently available systemic treatment options has proven BM preventive properties [1].

Considering the varying clinical courses, prognoses, and the considerable symptomatic burden, BM still remains a critical challenge in clinical oncology [2]. Consequently, prognostic indices have been established to guide treatment decisions in consideration of the prognosis [2].

Despite the high prevalence of neurological symptoms in patients diagnosed with BM from BC, the prognostic effect of the symptomatic burden in BM from BC has not been systematically explored so far [1]. However, modern patient cohorts underscore the significance of the symptom burden, as evidenced by findings from specific BM clinical trials in lung cancer, BC, and melanoma investigating novel targeted and immune-checkpoint inhibitor treatment modalities [3]. These trials, focusing on patients with asymptomatic BM not immediately requiring local therapy, have demonstrated noteworthy intracranial response rates for targeted and immunotherapeutic agents in the absence of additional local interventions [4–10].

In line with these results, the recent guidelines from the European Association of Neuro-Oncology and the European Society for Medical Oncology for the treatment of BM recommend the incorporation of symptomatic burden into treatment decisions. Specifically, the guidelines advocate the exploration of systemic treatment options in carefully selected patients with asymptomatic BM, particularly when a therapeutic agent with promising intracranial efficacy is available [11, 12].

Therefore, we aimed to explore the frequency of neurological symptoms at diagnosis of BM and evaluate its influence on the prognostic landscape in a unique real-world cohort of patients BC diagnosed between 1992 and 2020 as the foundation for future clinical trial planning and treatment decision-making.

## Methods

### Patients

Patients with BC BM treated at the Medical University of Vienna between 1992 and 2020 were included in this retrospective analysis (Supplementary Fig. 1). The Vienna Brain Metastasis Registry, an encompassing database documenting patients managed for brain-metastatic conditions at the Medical University from 1986 to 2023, served as the primary data source. All included patients were treated according to the respective best evidence at the time of treatment. The conduction of this study and the associated data collection and analyses were approved by the Ethics Committee of the Medical University of Vienna (vote 1692/2022).

### Clinical characteristics

If information regarding histopathological subtypes by immunohistochemical analysis and ERBB2 amplification was accessible, BC was further classified into hormone receptor-positive; HER2 (human epidermal growth factor receptor 2)-negative breast cancer (HR+ BC), HER2 overexpressing breast cancer (HER2+ BC), and triple-negative breast cancer (TN-BC). Notably, patients with leptomeningeal disease only or combined with BM were excluded from the analysis.

All included patients underwent magnetic resonance imaging (MRI) for the diagnosis of BM and received routine MRI re-stagings conducted approximately every 12 weeks after diagnosis of BM. While MRI is the gold standard imaging technique for symptomatic patients with suspected BM, screening MRI for asymptomatic patients was performed at the discretion of the clinical physician.

The diagnosis of the primary tumour and BM within 30 days was defined as the synchronous diagnosis of primary and metastatic lesions. Extracranial restaging was routinely performed every 2–4 months from the time of diagnosis of the primary tumour until death or last follow-up. Responses were evaluated according to the treating physician at the time of treatment. The disease status at the end-of-life period was investigated based on the last available imaging within 6 weeks before death. Intracranial progressive disease (PD) was defined as progressive BM without extracranial progression, extracranial PD was defined as extracranial progression in the absence of intracranial progression, and bicompartmental progression was defined as combined intra- and extracranial PD.

The breast cancer-specific Graded Prognostic Assessment (Breast-GPA) was calculated based on established prognostic parameters published previously [2].

Based on the obligatory and standardised documentation of patient’s data during clinical routine at the Medical University of Vienna, all data needed for this analysis were retrieved from medical records. In case of missing clinical data, the information is listed in the tables and Supplementary Material.

### Investigation of neurological symptoms

Clinical examination and history regarding neurological symptom burden were performed routinely at diagnosis of BM and documented in the patient’s record. In line with a recent study on neurological symptoms in patients with BM from lung cancer, patients in this analysis were categorised with a categorial variable as symptomatic (yes/no) if any neurological symptom including focal deficits, signs of increased intracranial pressure, epileptic seizures, or neuropsychological symptoms in relation to the diagnosed BM was documented in the patient’s data at baseline defined as the date of initial BM diagnosis [13].

In more detail, signs of increased intracranial pressure were defined as one or more of the following: headache, nausea, or emesis as published previously [14–16]. Neuropsychological symptoms were defined as cognitive dysfunction/impairment, or organic brain disorder. In the event of memory problems, episodes of new forgetfulness, or in the event of slow thinking and processing of information at the time of BM diagnosis, symptoms were summarised as cognitive dysfunction/impairment. In the event of hallucinations, delusions, personality changes, or delirium, symptoms were summarised as an organic brain disorder. Patients with neurological symptoms due to any underlying disease present before BM diagnosis were excluded from this analysis.

Supplementary Table 1 lists all documented neurological symptoms at diagnosis of BM.

Patients were classified as asymptomatic if they had no current neurological symptoms and had not experienced any prior neurological symptoms related to the present BM at diagnosis. Thus, patients whose asymptomatic status was influenced by the use of antiepileptic medication or steroids, due to prior neurological events, were not classified as asymptomatic. Notably, all prior neurological symptoms associated with the BM were consistently considered in the symptom classification process.

Given the retrospective design of the study, the Karnofsky performance status (KPS) remains the only quantitative measure available to approximate the impact of neurological symptoms on the patient’s condition.

Patients with no info on the neurological symptom status at BM diagnosis were excluded from the analysis (Supplementary Fig. 1).

### Statistical analysis

Brain metastases free survival (BMFS) was defined as the time from histological diagnosis of BC to radiological diagnosis of BM. Patients with a synchronous diagnosis of BC and BM were excluded from analysing the brain-metastatic-free survival period.

Overall survival (OS) was defined as the time from initial radiological diagnosis of BM to death or last follow-up. The Kaplan–Meier product-limit method was used to estimate OS. To estimate differences in OS between groups, the log-rank test was used.

Parameters according to neurological symptoms with a statistically significant association with survival prognosis in the univariate analysis were included together with the Breast-GPA in a multivariate analysis using the Cox proportional hazards model. Cox proportional hazard regression was used to estimate the hazard ratios. Ordinary nonparametric bootstrapping was used to investigate the stability of the COX proportional hazard model as part of the intern validation.

A two-tailed *p* value < 0.05 was considered to indicate statistical significance. Considering the exploratory and hypothesis-generating nature of this present study, no adjustment for multiple testing was applied [17].

Statistical analysis was performed using the Statistical Package for the Social Sciences (SPSS) version 28.0 software & R Version 4.3.1 (2023-06-16).

## Results

### Patient’s characteristics

A total of 6151 patients with BM from different solid tumours are included in the Vienna Brain Metastasis Registry. In total, 716/6151 (11.6%) patients with BM from BC and available information on the symptomatic status at diagnosis of BM were identified and included in this analysis (Supplementary Fig. 1). In 653/716 (91.2%) patients information on the receptor status was available including 260/653 (39.8%) patients with HR+ BC, 230/653 (35.2%) with HER2+ BC and 163/653 (25.0%) with TN-BC.

In total, 100/716 (14.0%) patients were diagnosed with Stage IV BC at time of primary tumour diagnosis. Of these, 20.0% (20/100) were synchronously diagnosed with primary tumour and BM. In patients with subsequent diagnosed BM, the median BMFS was 49 months (range 2–443 months).

See Table 1 and Supplementary Table 3 for further information on the clinical characteristics of the total patient cohort.

**Table 1:** Clinical characteristics of the entire cohort.

Clinical characteristics of the entire cohort (*n* = 716)		
	*n*	%
*Characteristics before BM diagnosis*		
Median age at BC diagnosis	50 (range 22–91)	
Stages I–III at diagnosis of BC	616	86.0
Stage IV at diagnosis of BC	100	14.0
Systemic therapy before BM diagnosis		
Performed	582	81.2
Median number of systemic therapylines prior to BM diagnosis	2 (range 1–10)	
Unknown	33	4.6
Radiotherapy of BC		
Performed	455	63.5
Unknown	40	5.6
Surgical resection of BC		
Performed	632	88.3
Unknown	12	1.7
*Characteristics at the time of BM diagnosis*		
Year of BM diagnosis		
1992–1999	21	2.9
2000–2009	293	40.9
2010–2022	402	56.1
Median age at BM diagnosis	56 (range 24–91)	
Median KPS at BM diagnosis	80 (range 10–100)	
Neurological symptoms at BM diagnosis		
Present	573	80.0
Focal deficits	505	70.5
Motor disorders	70	9.8
Hemiparesis	55	7.7
Ataxia	79	11.1
Cranial nerve palsy	165	23.0
Hypesthesia	73	10.3
Aphasia	75	10.6
Vertigo	261	36.8
Signs of increased intracranial pressure	275	48.0
Headache	208	29.1
Nausea and Emesis	165	23.0
Epileptic seizures	90	12.6
Focal seizures	37	5.2
Generalised seizures	43	6.0
Focal & generalised seizures	10	1.4
Neuropsychological symptoms	232	32.4
Organic brain disorder	38	5.4
Cognitive dysfunction/impairment	210	29.3
Absent	143	20.0
Corticosteroids intake at the time of BM diagnosis		
Present	231	32.3
Absent	462	64.5
Unknown	23	3.20
Status of BC at BM diagnosis		
Complete response after systemic therapy	10	1.4
Stable disease	199	27.8
Partial response	40	5.6
Progressive disease	262	36.6
No evidence of disease after surgical resection	156	21.8
Unknown	29	4.1
Synchronous diagnosis of BM and BC	20	2.8
Median time to diagnosis of BM in subsequent diagnosed patients (in months)	49 (range 2–443)	
Median time to diagnosis of BM in Stages I–III patients (in months)	53 (range 2–443)	
Median time to diagnosis of BM in Stage IV patients (in months)	22 (range 3–144)	
Extracranial metastases at BM diagnosis		
Present	553	77.2
Absent	163	22.8
Number of BM at diagnosis		
1	261	36.5
2–3	204	28.5
≥4	251	35.1
Size of BM at diagnosis		
≥3 cm	180	25.1
<3 cm	536	74.9
Localisation of BM at diagnosis		
Supratentorial	310	43.3
Infratentorial	102	14.2
Both	304	42.5
Localisation side of BM at diagnosis		
Right	161	22.5
Left	188	26.3
Both	367	51.2
*Characteristics after the time of BM diagnosis*		
Initial treatment strategy after BM diagnosis		
Focal radiotherapy	221	30.9
WBRT	231	32.3
WBRT + focal radiotherapy	62	8.7
Neurosurgical resection	88	12.3
Neurosurgical resection + focal radiotherapy	11	1.5
Neurosurgical resection + WBRT	38	5.3
Neurosurgical resection + WBRT + focal radiotherapy	2	0.3
Neurosurgical resection + radiation of resection cavity	21	2.9
Neurosurgical resection + radiation of resection cavity + focal radiotherapy	5	0.7
Systemic treatment	13	1.8
Median number of systemic therapylines after BM diagnosis	2 (range 1–5)	
BSC	24	3.4
Intracranial progression after initial treatment strategy at BM diagnosis		
Present	396	55.3
Absent	320	44.7
Median time from diagnosis of BM to intracranial progression	7 (range 1–125)	
Extracranial progression after initial treatment strategy at BM diagnosis		
Present	294	41.1
Absent	422	58.9
Median time from diagnosis of BM to extracranial progression	5 (range 1–201)	
Status at last follow-up		
Diseased	606	84.6
Status at the end-of-life period (in diseased patients)		
Intracranial progression	82	11.5
Extracranial progression	104	14.5
Combined progression	181	25.3
Unknown	349	48.7
Alive	110	15.4
Median OS from diagnosis of BM	9 (range 1–212)	
Median OS from diagnosis of BC	60 (range 1–444)	

*BC* breast cancer, *BM* brain metastases, *BSC* best supportive care, HR+ BC: hormone receptor-positive(HR)/human epidermal growth factor receptor 2 (HER2)-negative breast cancer, *HER2*+ *BC* HER2 overexpressing BC, *KPS* Karfnofsky Performance Scale, *OS* overall survival, *TN-BC* triple-negative breast cancer, *WBRT* whole brain radiation therapy.

### Neurological symptom burden at diagnosis of BM

**Fig. 1:**
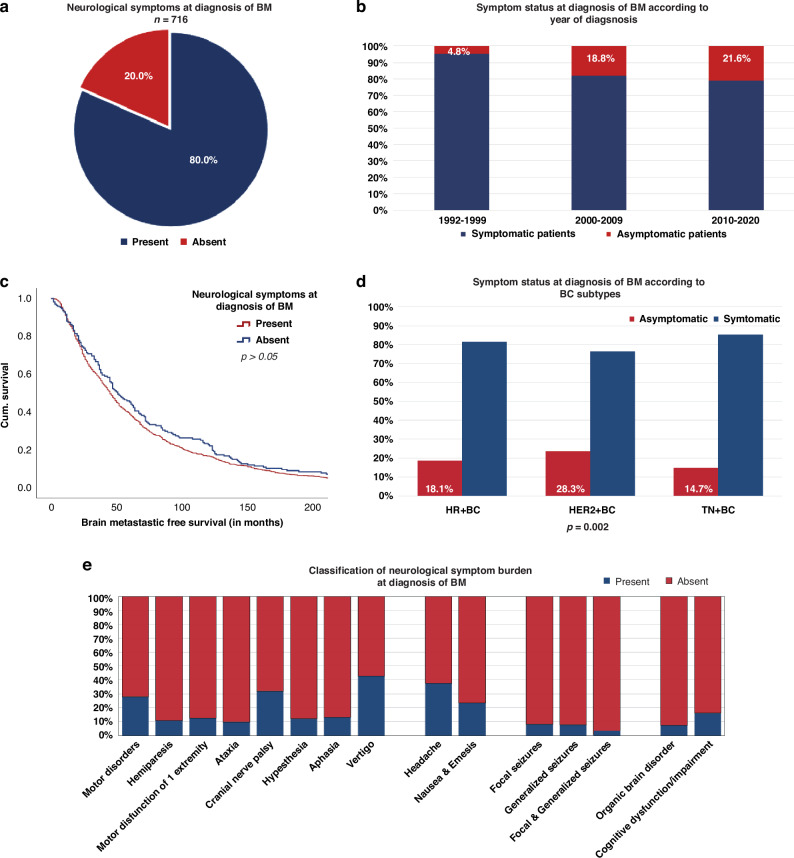
Neurological symptom burden in patients with BM from BC. Presence of the neurological symptoms at diagnosis of BM (**a** + **e**) according to the year of BM diagnosis (**b**), BMFS (**c**), and subtypes of BC (**d**).

In total, 80% (573/716) of the patients presented with neurological symptoms at the time of BM diagnosis (Fig. 1a). A numerical increase of asymptomatic patients at the time of BM diagnosis was observed over the last three decades (1992–1999: 4.8% vs. 2010–2020: 21.6%; *p* = 0.135; Table 2; Fig. 1b)—irrespective of the underlying BC subtype.

**Table 2:** Clinical characteristics according to the neurological symptomatic status of the total cohort (*n* = 716).

Neurological symptoms at BM diagnosis					
	Present (*n* = 573)		Absent (*n* = 143)		*p* value
	*n*	%	*n*	%	
*Characteristics at the time of BM diagnosis*					
Years of diagnosis					
1992–1999	20	3.5	1	0.7	0.135
2000–2009	238	41.5	55	38.5	
2010–2020	315	55.0	87	60.8	
Median age at BM diagnosis	56 (range 24–91)		56 (range 29–85)		0.434
Median KPS at diagnosis	80 (range 10–100)		90 (range 40–100)		0.598
Extracranial metastases at BM diagnosis					
Presence	432	75.4	121	84.6	***0.019***
Absence	141	24.6	22	15.4	
Synchronous diagnosis of BC and BM	14	2.4	6	4.2	0.225
Subsequent diagnosis of BM	559	97.6	137	95.8	
Median time to diagnosis of BM (months)	48 (2–443)		49 (2–333)		0.720
Median time to diagnosis of BM in Stages I–III patients (in months)	53 (4–443)		53 (2–290)		0.611
Median time to diagnosis of BM in Stage IV patients (in months)	22 (4–126)		18 (3–144)		0.861
Number of BM at diagnosis					
1	212	37.0	49	34.3	0.815
2–3	161	28.1	43	30.1	
≥4	200	34.9	51	35.7	
Size of BM at diagnosis					
≥3 cm	203	35.4	23	16.1	***<0.001***
<3 cm	370	64.6	120	83.9	
Localisation of BM					
Supratentorial	251	43.8	59	41.3	0.621
Infratentorial	85	14.8	17	11.8	
Both	237	41.4	67	46.9	
Localisation side of BM					
Right	132	23.0	32	22.4	0.353
Left	160	27.9	32	22.4	
Both	281	49.4	79	55.2	
Corticosteroids intake at BM diagnosis (only patients with known status included)					
Presence	178	31.1	53	37.1	0.258
Absence	373	65.1	89	62.2	
*Characteristics after the time of BM diagnosis*					
Initial treatment strategy after BM diagnosis					
Focal radiotherapy	170	32.9	51	37.5	***0.040***
WBRT	190	36.8	41	30.1	
WBRT + focal radiotherapy	57	11.0	5	3.7	
Neurosurgical resection	72	13.9	16	11.8	
Neurosurgical resection + focal radiotherapy	8	1.5	3	2.2	
Neurosurgical resection + WBRT	31	6.0	7	5.1	
Neurosurgical resection + WBRT + focal radiotherapy	2	0.4	0	0	
Neurosurgical resection + radiation of resection cavity	17	3.3	4	2.9	
Neurosurgical resection + radiation of resection cavity + focal radiotherapy	5	1.0	0	0	
Systemic treatment	5	1.0	8	5.9	
BSC	16	3.1	8	5.9	
Intracranial progression after BM diagnosis					
Present	257	44.9	63	44.1	0.864
Absent	316	55.1	80	55.9	
Median time from diagnosis of BM to intracranial progression	15 (range 4–18)		17 (range 8–19)		0.273
Extracranial progression after BM diagnosis					
Present	226	39.4	77	53.8	0.060
Absent	347	60.5	66	46.1	
Median time from diagnosis of BM to extracranial progression	5 (range 4–5)		6 (range 3–8)		***0.008***
Disease status at the end-of-life period (available in 338)					
Intracranial progression	64	11.1	18	12.9	0.788
Extracranial progression	79	13.7	25	17.5	
Combined progression	123	21.1	29	20.3	
Median OS from diagnosis of BM	9 (range 1–212)		20 (range 1–146)		***<0.001***

*BC* breast cancer, *BM* brain metastases, *BSC* best supportive care, *HR-BC* HER2 (human epidermal growth factor receptor 2)-negative breast cancer, *HER2-BC* HER2 overexpressing breast cancer, *KPS* Karfnofsky Performance Scale, *OS* overall survival, *TN-BC* triple-negative breast cancer, *WBRT* whole brain radiation therapy.

Bold and italic numbers highlight statistical significant results.

Significant differences according to symptomatic burden at BM diagnosis were observed between BC subtypes. Patients with HER2+ BC were more frequently diagnosed with asymptomatic BM during staging procedures (65/230;28.3%), compared to HR+ BC (47/260; 18.1%) and TN-BC (24/163; 14.7%; chi-square test; *p* = 0.002; Supplementary Table 2; Fig. 1d).

#### Focal deficits

Focal deficits were the most commonly documented neurological symptoms at the time of BM diagnosis (88.0%; 504/573; Fig. 1e). According to BC subtypes, patients with TN-BC (133/139; 95.7%) presented most frequently with focal deficits compared to HR+ BC (187/213; 87.8%) and HER2+ BC (136/165; 82.4%; *p* = 0.005; chi-square test; Supplementary Table 3).

#### Signs of increased intracranial pressure

In total, 275/573 (48.0%) patients showed signs of increased intracranial pressure at the time of BM diagnosis (Fig. 1e). No differences were observed according to BC subtypes (*p* > 0.05; chi-square test; Supplementary Table 3).

#### Epileptic seizures

In total, 15.7% (90/573) of symptomatic patients had an epileptic event at BM diagnosis. Of these, 42.2% (38/90) presented with focal seizures, 46.7% (42/90) with generalised seizures, and 11.1% (10/90) with combined focal and generalised seizures at the time of BM diagnosis (Fig. 1e). No significant differences were observed according to BC subtypes regarding the frequency of seizures at baseline (*p* > 0.05; chi-square test; Supplementary Table 3).

#### Neuropsychological symptoms

Neuropsychological symptoms were present in 31.9% (183/573) of symptomatic patients at baseline. Cognitive impairment/dysfunction (151/183; 82.5%) was most frequently documented, followed by organic brain syndrome (32/183; 17.5%), irrespective of the underlying BC subtype (*p* > 0.05; chi-square test; Supplementary Table 3; Fig. 1e).

See additional information on symptoms at BM diagnosis in Table 1, Supplementary Table 3, and Fig. 1e.

### Clinical characteristics at diagnosis of BM associated with neurological symptom burden

BMFS was not statistically significantly different between patients with asymptomatic and symptomatic BM (48 vs. 49 months; *p* = 0.720; log-rank test, Table 2, Fig. 1c).

**Fig. 2:**
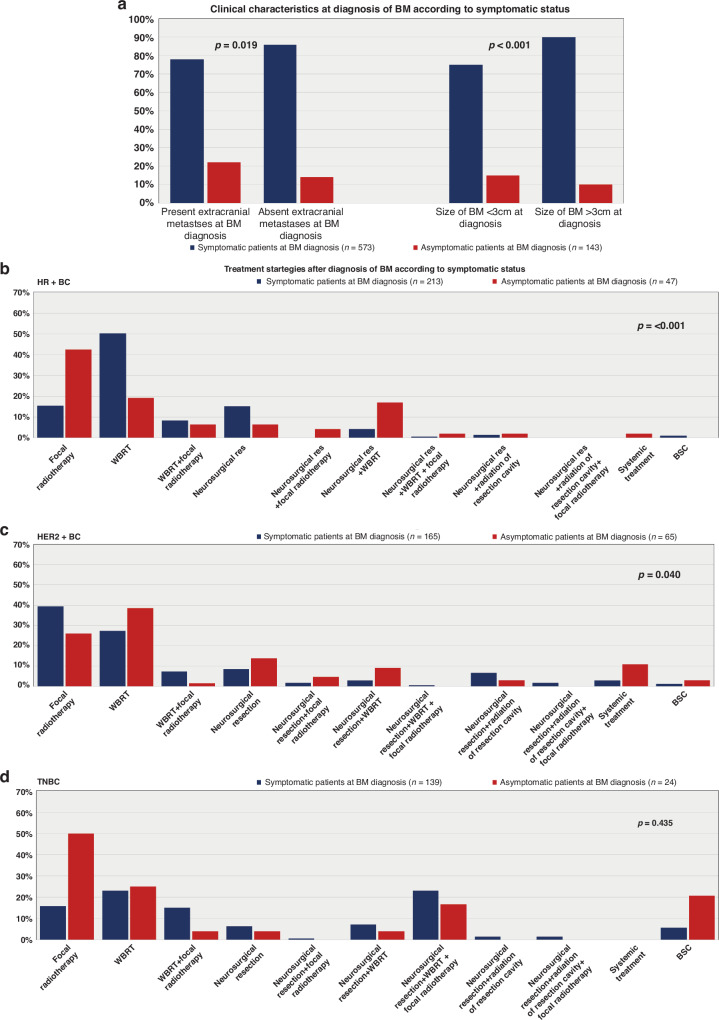
Presentation at diagnosis of BM and further clinical course according to the neurological symptom status. Clinical characteristics at BM diagnosis of BM in symptomatic and asymptomatic patients (**a**); treatment strategies after BM diagnosis in HR+ BC (**b**), HER2+ BC (**c**) and TN-BC (**d**) patients according to symptomatic status.

Patients with BM as the only metastatic site had a significantly higher rate of symptomatic disease compared to patients with known extracranial metastasis (*p* = 0.019; chi-square test; Table 2; Fig. 2a). This effect was most pronounced in HER2+ BC, as 83.7% (41/49) of patients with absent extracranial metastases showed neurological symptoms at diagnosis of BM compared to 68.5% (124/181) of patients with concurrent BM and extracranial disease (*p* = 0.036; chi-square test; Supplementary Table 2).

Furthermore, the size of BM at diagnosis was also significantly associated with neurological symptoms. A total of 92.6% (162/175) of patients with BM ≥ 3 cm showed neurological symptoms, while only 7.4% (13/175) of patients with BM < 3 cm were symptomatic at BM diagnosis (*p* < 0.001; chi-square test; Table 2; Fig. 2a). In particular, 92.2% of (47/51) patients with HER2+ BC and BM ≥ 3 cm presented with neurological symptoms compared to 64.5% (98/152) with BM < 3 cm (*p* < 0.001; chi-square test; Supplementary Table 2).

In contrast, the number of BM or the localisation (hemispheres/lobes) as well as the age and KPS of the patients at diagnosis of BM were not associated with the neurological burden (*p* > 0.05; chi-square test; Table 2; Supplementary Table 2).

All additional information on the clinical characteristics associated with the symptomatic burden at BM diagnosis is given in Table 3, Supplementary Table 2, and Fig. 2a.

### Treatment strategies after diagnosis of BM according to neurological symptom burden

Radiotherapy, encompassing whole brain radiation therapy (WBRT) and focal radiotherapy presented as the predominant therapeutic modality following the diagnosis of BM in BC patients across the decades (Supplementary Table 3). However, we observed noteworthy variations in initial treatment strategies upon the symptomatology and BC subtype. Specifically, among symptomatic patients with HR+ BC, WBRT was the predominant choice (50.2%; 107/213), surpassing focal radiotherapy alone (15.5%; 33/213; *p* < 0.001; chi-square test; Supplementary Table 2; Fig. 2d).

In total, 14.6% (31/213) of HR+ BC patients with neurological symptoms received combinational approaches after diagnosis of BM: 18/31 (58.1%) underwent WBRT along with focal radiotherapy, while 13/31 patients (41.9%) received additional radiation therapy after neurosurgery at the time of BM diagnosis.

Conversely, symptomatic HER2+ BC patients predominantly underwent focal radiotherapy (39.4%; 65/165) alone at diagnosis of BM. A smaller subset (7.3%; 12/165) underwent additional WBRT following focal radiotherapy. WBRT as the sole initial treatment was chosen in 27.3% (45/165) of patients (*p* = 0.040, chi-square test; see Supplementary Table 2; Fig. 2c). Additionally, 13.9% (23/165) of symptomatic HER2+ BC patients received further radiotherapy after undergoing initial neurosurgery at BM diagnosis.

In asymptomatic patients, regardless of the underlying BC subtype, SRS consistently emerged as the most frequently employed treatment modality over the decades (Supplementary Table 4). Moreover, a discernible upward trend in the utilisation of systemic therapy as an initial treatment approach after diagnosis of BM was noted in asymptomatic patients across different decades (1992–1999: 0% vs. 2010–2020: 5.6%; Supplementary Table 4).

In the cohort of TN-BC patients, no significant differences in treatment approaches were found based on neurological symptoms at the time of BM diagnosis (*p* > 0.05; chi-square test; Supplementary Table 2; Fig. 2a).

**Fig. 3:**
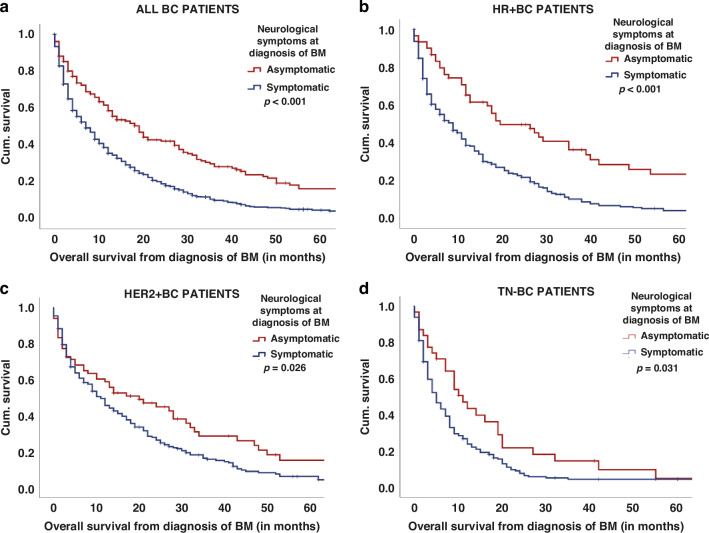
Impact of neurological symptoms on the survival prognosis of BM from BC. Overall survival from diagnosis of BM according to the neurological symptom status at BM diagnosis in all patients (**a**) as well as in HR+ BC (**b**), HER2+ BC (**c**), and TN-BC (**d**) patients.

Furthermore, no variations in terms of intracranial recurrence patterns or the end-of-life period concerning neurological symptom status have been observed. However, patients diagnosed with BM in an asymptomatic state exhibited a significantly prolonged median duration to extracranial progression compared to symptomatic patients (6 vs. 5 months; *p* = 0.008; log-rank test, Table 2, and Supplementary Table 2). Find a comprehensive overview in Table 2, Supplementary Table 2, and Figs. 2a–3f on therapeutic modalities, patterns of intra- and extracranial progression, and the end-of-life period in accordance with the neurological symptom burden.

### Association of neurological symptoms with survival prognosis

Patients without neurological symptoms at baseline had a significantly prolonged median OS from diagnosis of BM compared to symptomatic patients (20 vs. 9 months, *p* < 0.001; log-rank test; Table 2; Fig. 3a). This observed effect persisted consistently across all BC subtypes (HR+ BC 29 vs. 9 months; HER2+ BC 24 vs. 12 months; TN 12 vs. 6 months; *p* < 0.05; log-rank test; Supplementary Table 2; Fig. 3b–d) and throughout the three decades (1992–1999: 14 vs. 9 months; 2000–2009: 19 vs. 8 months; 2010–2020: 27 vs. 9 months; *p* < 0.05; log-rank test, Supplementary Table 4).

Subsequently, we analysed the prognosis based on the type of neurological symptom. Here, patients with neuropsychological symptoms presented a significantly shorter median OS compared to patients with other neurological symptoms (12 vs. 5 months; *p* < 0.001; log-rank test; Supplementary Table 7). Specifically, the presence of cognitive dysfunction at the time of BM diagnosis was correlated with a substantially compromised median OS in contrast to patients presenting with other neurological symptoms (12 vs. 6 months; *p* < 0.001; log-rank test; Supplementary Table 7).

### Multivariate survival analysis including the symptomatic burden and established prognostic scores

To assess the prognostic independence of neurological symptoms in relation to other prognostic factors, we incorporated parameters such as Breast-GPA, the size of BM at diagnosis, treatment strategies (including WBRT, SRS, neurosurgery, systemic therapy, BSC), and neurological symptoms into a multivariate model. In this analysis, the presence of neurological symptoms (HR: 1.6; 95% CI, 1.35–1.95; *p* < 0.001; Supplementary Table 6A), the treatment approach after BM diagnosis (HR: 1.1; 95% CI, 1.03–1.17; *p* = 0.04) and the Breast-GPA (HR: 1.4; 95% CI, 1.27–1.49; *p* < 0.001; Cox-regression model; Supplementary Table 6A) remained independently associated with prognosis. In addition, we performed an intern validation with the bootstrapping method to prove our analysis. In this, the results of the bootstrapping indicated that our Cox-regression model is stable as consistent hazard ratio estimates across different subsets of the data (Supplementary Table 6B).

Furthermore, the impact of neurological symptoms on survival prognosis was irrespective of the Breast-GPA classes, as asymptomatic patients in good (2.5–4.0 points) as well as in lower (0.0–2.0 points) prognostic classes showed an improved median OS compared to symptomatic patients (*p* < 0.05; log-rank test; Supplementary Table 6).

## Discussion

BM presents a frequent and dreaded complication of BC. Over the last decades, CNS screening in frequently BM-causing entities led to higher detection of BM in asymptomatic settings and, thereby, raised the question of a symptom-adapted therapy approach [1]. In line, previous studies revealed prolonged OS in patients with asymptomatic BM from small and non-small lung cancer (NSCLC) as well as melanoma, compared to symptomatic BM [13, 18, 19]. To date, no studies on the prognostic impact of neurological symptoms in patients with BM from BC have been performed. This study is—to the authors’ best knowledge—the first study identifying a strong and independent prognostic value of the neurological symptomatic burden at diagnosis of BM in patients with advanced BC, strongly supporting the incorporation of the symptomatic burden in a prognosis-adapted treatment approach.

Due to the high likelihood of CNS involvement in melanoma and lung cancer, screening of the CNS has consequently become a recommended procedure in Stages II–IV NSCLC, small cell lung cancer at any stage, and Stage IV melanoma [11, 20, 21]. In BC, screening for BM in BC is not recommended, however, suggested in patients with metastatic HER2+ BC and TN-BC [11, 22]. In line with the current guidelines, only ~20% of patients with BC were diagnosed with asymptomatic BM in our cohort. In contrast, over 30% of patients with NSCLC and, around half of the patients with melanoma were diagnosed with asymptomatic BM during screening and staging procedures in two previous studies [13, 18]. A recent American retrospective study aimed to identify the potential value of brain-directed MRI screening in BC patients diagnosed between 2000 and 2015 by comparing the presentation, intracranial management, and outcome between patients with BM from BC (which are largely not screened for BM) and from NSCLC (which are largely screened) [23]. In this study, BC patients presented with more advanced intracranial disease than patients with NSCLC, and consequently required more frequent WBRT as initial management. Interestingly, after initial brain-directed therapy, no differences in recurrence or salvage therapy-based outcomes were observed between the two cohorts. However, the survival results have to be interpreted with caution as the BC cohort included a relatively high number of patients who were screened for BM within the scope of clinical trials or provider preferences compared to previous studies. Furthermore, in contrast to our results, no focus was given on the presence of neurological symptoms at diagnosis of BM and its impact on the further clinical course in this study. Importantly, our retrospective data are the first to present an independent association of the symptom burden at diagnosis of BM and the median OS in a large real-life cohort of patients with BM from BC. The fact that we found no significant results between the BMFS between asymptomatic and symptomatic patients—reflecting no lead time bias in our results—strongly supports further investigations on BM screening in modern BC cohorts. Indeed, prospective clinical trials are urgently needed to define BC patients who benefit the most from CNS screening. Even though we assume that primarily patients with existing metastases would benefit from CNS screening, our retrospective data analysis indicated that specific high-risk groups, such as HER2+ BC patients could also benefit from screening in early stages in the absence of extracranial disease. Defining these cohorts is essential to optimise resource use and support healthcare systems, as MRIs are costly and not always available in clinical routine. By identifying specific high-risk groups, screening could be recommended to the defined limited cohort of patients, thereby reducing costs for the healthcare systems and making screening more feasible. In line with this, our retrospective study provides valuable information which can support the foundation for defining these cohorts in future prospective trials.

Neurological symptoms have also gained relevance as part of the decision-making processes in BM treatment over the last few years. Several clinical trials presented surprising intracranial response rates of targeted—and immunotherapy in advanced, brain-metastatic cancer—specifically in asymptomatic patients [3]. Specifically, HER2+ BC patients can be introduced to several CNS-effective systemic treatment approaches [24]. These emerging therapeutic breakthroughs in HER2+ BC over the last years may also explain the higher detection of asymptomatic BM during screening and staging procedures in HER2+ BC in our cohort. Importantly, systemic treatment approaches may allow delaying WBRT, and thereby increase the quality of life—the major therapeutic aim in patients with advanced cancer in palliative settings. Given the frequent development of new targeted and immune-modulating therapies, our data strongly support the further evaluation in BM-specific trials and the ideal combination/sequencing of systemic and local therapies.

Indeed, the reduction of long-term side effects presents an important therapeutic goal in brain-metastatic BC patients, as these patients present the most favourable median OS after diagnosis of BM up to 15 months and longer compared to other BM-causing primary entities [1]. In our cohort, the diagnosis of BM in asymptomatic settings further improved the median OS, irrespectively of the underlying BC subtype and independently from other prognostic factors. However, so far, neurological symptoms are not included in the prognostic assessment of patients with BM from BC. Therefore, our data strongly support further prospective investigation to incorporate the symptomatic burden in the prognostic assessment of newly diagnosed BC BM patients.

While our study presents the most extensive real-world cohort focusing on the incidence of neurological symptoms at diagnosis of BM and its subsequent impact on the clinical course in BC patients, certain limitations must be considered in interpreting the findings. The long inclusion period from 1992 to 2020 may lead to potential bias, given the conceivable alterations in imaging and treatment tools over this extended duration. Nonetheless, concerted efforts were made to reduce these potential biases. On the one hand, exclusively those patients with MRI at BM diagnosis were included, aiming to enhance the diagnostic homogeneity of our findings and ensure that our analysis reflects modern BC BM cohorts, as MRI is the current gold standard for BM diagnosis. Furthermore, information regarding neurological symptoms was documented comprehensively in the patient’s records. Nevertheless, the predominant limitation lies in the retrospective design of this study. However, the encouraging findings from our study could serve as a basis for subsequent prospective trials utilising quantitative methodologies, such as the preliminary validated Neurologic Assessment in Neuro-Oncology (NANO) scale, to systematically evaluate the symptomatic burden in BC BM [25]. The NANO scale was designed to be easy to perform also by non-neurologists, focusing on the key aspects of neurological function. As a standardised tool, the NANO scale allows the tracking of changes compared to a previous baseline, which may offer valuable insights into the progression of symptom burden throughout the clinical course of the disease [26]. This would be essential for drawing meaningful conclusions to guide clinical decision-making.

In addition, the analysis over three decades provides a distinctive opportunity to gain a more profound understanding of the incidences, characteristics, and prognostic value of the neurological symptom burden in patients with BM BC over the past 30 years.

In conclusion, this is the first study that includes a detailed characterisation of the neurological symptom burden at diagnosis of BM across all subtypes of BC. More importantly, our study identified the presence of neurological symptoms at diagnosis of BM as an independent and strong prognostic factor in a large, distinctive real-world cohort of patients with brain-metastatic BC. The outcomes underscore the critical need for subsequent prospective trials to thoroughly examine the integration of neurological symptom burden into prognostic assessments for individuals with BM originating from BC.

## Supplementary information

Supplementary Table 1

Supplementary Table 2

Supplementary Table 3

Supplementary Table 4

Supplementary Table 5

Supplementary Table 6

Supplementary Table 7

Supplementary Figure 1

## Data Availability

The datasets generated and analysed during the current study are available from the corresponding author upon reasonable request.
