# Supplementary material for: Prognostic relevance of the neurological symptom burden in brain metastases from breast cancer
Source: Br J Cancer. 2025 Mar 1;132(8):733–43. doi: 10.1038/s41416-025-02967-w (PMC11997164; doi:10.1038/s41416-025-02967-w)
Supplement: Supplementary file 1 — Supplementary Table 1 [file 41416_2025_2967_MOESM1_ESM.docx]

**Supplementary Table 1:** Neurological symptoms evaluated at diagnosis of BM

| **NEUROLOGICAL SYMPTOMS AT BM DIAGNOSIS** | | | |
| --- | --- | --- | --- |
| Focal deficits | Signs of increased intracranial pressure | Epileptic seizures | Neuropsychological symptoms |
| Ataxia | Headache | Generalized seizures | Cognitive dysfunction/ |
| Vertigo | Nausea & Emesis | Focal seizure | impairment |
| Motor disorders |  |  | Organic brain disorder |
| Hemiparesis |  |  |  |
| Cranial nerve palsy |  |  |  |
| Hypesthesia |  |  |  |
| Aphasia |  |  |  |

**Abbreviations:** BM: Brain metastases
