# Supplementary material for: Prognostic relevance of the neurological symptom burden in brain metastases from breast cancer
Source: Br J Cancer. 2025 Mar 1;132(8):733–43. doi: 10.1038/s41416-025-02967-w (PMC11997164; doi:10.1038/s41416-025-02967-w)
Supplement: Supplementary file 2 — Supplementary Table 2 [file 41416_2025_2967_MOESM2_ESM.docx]

**Supplementary Table 2:** Clinical characteristics according to neurological symptoms in BC subtypes

| **NEUROLOGICAL SYMPTOMS AT BM DIAGNOSIS according to BC subtype** | | | | | |
| --- | --- | --- | --- | --- | --- |
|  | **Present**  (n= 517) | | **Absent**  (n=136) | | **p-value** |
| **CHARACTERISTICS AT TIME OF BM DIAGNOSIS** | | | | | |
| **Neurological symptoms at BM diagnosis** | | | | | |
| HR+BC | 213 | 37.1 | 47 | 32.9 | ***0.002*** |
| HER2+BC | 165 | 28.8 | 65 | 45.5 |  |
| TN-BC | 139 | 24.2 | 24 | 16.8 |  |
| Unknown receptor status | 56 | 9.8 | 7 | 4.9 |  |
| **Year of BM diagnosis** | | | | | |
| **1992-1999** |  |  |  |  |  |
| HR+BC | 7 | 1.4 | 0 | 0 | 0.451 |
| HER2+BC | 1 | 0.2 | 0 | 0 | 0.191 |
| TN-BC | 5 | 1.0 | 0 | 0 | 0.474 |
| **2000-2009** |  |  |  |  |  |
| HR+BC | 78 | 15.1 | 18 | 13.2 | 0.451 |
| HER2+BC | 75 | 14.5 | 25 | 18.4 | 0.191 |
| TN-BC | 50 | 9.7 | 7 | 5.1 | 0.474 |
| **2010-2020** |  |  |  |  |  |
| HR+BC | 128 | 24.8 | 29 | 21.3 | 0.451 |
| HER2+BC | 90 | 17.4 | 39 | 28.7 | 0.191 |
| TN-BC | 84 | 16.2 | 17 | 12.5 | 0.474 |
| **Median age at BM diagnosis** | | | | | |
| HR+BC | 59 (range 24-91) | | 56 (range 29-78) | | 0.516 |
| HER2+BC | 53 (range 27-85) | | 57 (range 34-85) | | 0.650 |
| TN-BC | 51 (range 26-79) | | 57 (range 33-71) | | 0.714 |
| **Median KPS at diagnosis** | | | | | |
| HR+BC | 80 (range 10-100) | | 90 (range 60-100) | | 0.067 |
| HER2+BC | 80 (range 30-100) | | 80 (range 40-100) | | 0.345 |
| TN-BC | 80 (range 30-100) | | 80 (range 50-100) | | 0.784 |
| **Extracranial metastases at BM diagnosis** | | | | | |
| **Presence** |  |  |  |  |  |
| HR+BC | 178 | 34.4 | 40 | 29.4 | 0.795 |
| HER2+BC | 124 | 24.0 | 57 | 41.9 | 0.036 |
| TN-BC | 92 | 17.8 | 19 | 14.0 | 0.208 |
| **Absence** |  |  |  |  |  |
| HR+BC | 35 | 6.8 | 7 | 5.1 | 0.795 |
| HER2+BC | 41 | 7.9 | 8 | 5.9 | 0.036 |
| TN-BC | 47 | 9.1 | 5 | 3.7 | 0.208 |
| **Synchronous diagnosis of BC and BM** | | | | | |
| HR+BC | 6 | 1.2 | 3 | 2.2 | 0.226 |
| HER2+BC | 3 | 0.6 | 2 | 1.5 | 0.556 |
| TN-BC | 4 | 0.8 | 0 | 0.0 | 0.400 |
| **Subsequent diagnosis of BM** | | | | | |
| HR+BC | 207 | 40.0 | 44 | 32.4 | 0.226 |
| HER2+BC | 162 | 31.3 | 63 | 46.3 | 0.556 |
| TN-BC | 135 | 26.1 | 24 | 17.6 | 0.400 |
| **Median time to diagnosis of BM** (in months) | | | | | |
| HR+BC | 77 (4-365) | | 97 (2-277) | | 0.890 |
| HER2+BC | 41 (4-275) | | 45 (4-290) | | 0.890 |
| TN-BC | 32 (2-298) | | 21 (2-333) | | 0.890 |
| **Number of BM at diagnosis** | | | | | |
| **1** | | | | | |
| HR+BC | 92 | 17.8 | 16 | 11.8 | 0.061 |
| HER2+BC | 56 | 10.8 | 20 | 14.7 | 0.230 |
| TN-BC | 39 | 7.5 | 11 | 8.1 | 0.197 |
| **2-3** | | | | | |
| HR+BC | 58 | 11.2 | 21 | 15.4 | 0.061 |
| HER2+BC | 52 | 10.1 | 15 | 11.0 | 0.230 |
| TN-BC | 38 | 7.4 | 4 | 2.9 | 0.197 |
| ≥4 | | | | | |
| HR+BC | 63 | 12.2 | 10 | 7.4 | 0.061 |
| HER2+BC | 57 | 11.0 | 30 | 22.1 | 0.230 |
| TN-BC | 62 | 12.0 | 9 | 6.6 | 0.197 |
| **Size of BM at diagnosis** | | | | | |
| **≥3cm** | | | | | |
| HR+BC | 66 | 12.8 | 15 | 11.0 | ***0.027*** |
| HER2+BC | 51 | 9.9 | 7 | 5.1 | ***<0.001*** |
| TN-BC | 60 | 11.6 | 6 | 4.4 | 0.089 |
| **<3cm** | | | | | |
| HR+BC | 131 | 25.3 | 48 | 35.3 | ***0.027*** |
| HER2+BC | 108 | 20.9 | 64 | 47.1 | ***0.001*** |
| TN-BC | 81 | 15.7 | 16 | 11.8 | 0.089 |
| **Localization of BM** | | | | | |
| **supratentorial** | | | | | |
| HR+BC | 108 | 20.9 | 24 | 17.6 | 0.437 |
| HER2+BC | 47 | 9.1 | 23 | 16.9 | 0.407 |
| TN-BC | 69 | 13.3 | 9 | 6.6 | 0.489 |
| **infratentorial** | | | | | |
| HR+BC | 27 | 5.2 | 2 | 1.5 | 0.437 |
| HER2+BC | 38 | 7.4 | 10 | 7.4 | 0.407 |
| TN-BC | 16 | 3.1 | 4 | 2.9 | 0.489 |
| both | | | | | |
| HR+BC | 80 | 15.5 | 19 | 14.0 | 0.437 |
| HER2+BC | 80 | 15.5 | 32 | 23.5 | 0.407 |
| TN-BC | 53 | 10.3 | 12 | 8.8 | 0.489 |
| **Localization side of BM** | | | | | |
| **right** | | | | | |
| HR+BC | 56 | 10.8 | 10 | 1.9 | 0.318 |
| HER2+BC | 34 | 6.6 | 16 | 3.1 | 0.601 |
| TN-BC | 31 | 6.0 | 5 | 1.0 | 0.968 |
| **left** | | | | | |
| HR+BC | 67 | 13.0 | 10 | 1.9 | 0.318 |
| HER2+BC | 42 | 8.1 | 13 | 2.5 | 0.601 |
| TN-BC | 37 | 7.2 | 7 | 1.4 | 0.968 |
| **both** | | | | | |
| HR+BC | 92 | 17.8 | 25 | 18.4 | 0.318 |
| HER2+BC | 89 | 17.2 | 36 | 26.5 | 0.601 |
| TN-BC | 70 | 13.5 | 13 | 9.6 | 0.968 |
| **CHARACTERISTICS AFTER TIME OF BM DIAGNOSIS** | | | | | |
| **Initial treatment strategy after BM diagnosis** | | | | | |
| **Focal radiotherapy*** | | | | | |
| HR+BC | 33 | 6.3 | 20 | 14.7 | ***<0.001*** |
| HER2+BC | 65 | 12.6 | 17 | 12.5 | ***0.040*** |
| TN-BC | 22 | 4.3 | 12 | 8.8 | 0.435 |
| **WBRT** | | | | | |
| HR+BC | 107 | 20.7 | 9 | 6.6 | ***<0.001*** |
| HER2+BC | 45 | 0.8 | 25 | 18.4 | ***0.040*** |
| TN-BC | 32 | 6.2 | 6 | 4.1 | 0.435 |
| **WBRT + focal radiotherapy** | | | | | |
| HR+BC | 18 | 3.5 | 3 | 2.2 | ***<0.001*** |
| HER2+BC | 12 | 2.3 | 1 | 0.7 | ***0.040*** |
| TN-BC | 21 | 4.1 | 1 | 0.7 | 0.435 |
| **Neurosurgical resection** | | | | | |
| HR+BC | 32 | 6.2 | 3 | 2.2 | ***<0.001*** |
| HER2+BC | 14 | 2.7 | 9 | 6.6 | ***0.040*** |
| TN-BC | 9 | 1.7 | 1 | 0.7 | 0.435 |
| **Neurosurgical resection + focal radiotherapy** | | | | | |
| HR+BC | 0 | 0 | 2 | 1.5 | ***<0.001*** |
| HER2+BC | 3 | 0.6 | 3 | 2.2 | ***0.040*** |
| TN-BC | 1 | 0.2 | 0 | 0 | 0.435 |
| **Neurosurgical resection + WBRT** | | | | | |
| HR+BC | 9 | 1.7 | 8 | 5.8 | ***<0.001*** |
| HER2+BC | 5 | 1.0 | 6 | 4.1 | ***0.040*** |
| TN-BC | 10 | 1.9 | 1 | 0.7 | 0.435 |
| **Neurosurgical resection + WBRT + focal radiotherapy** | | | | | |
| HR+BC | 1 | 0.2 | 1 | 0.7 | ***<0.001*** |
| HER2+BC | 1 | 0.2 | 0 | 0 | ***0.040*** |
| TN-BC | 32 | 6.1 | 4 | 2.9 | 0.435 |
| **Neurosurgical resection + radiation of resection cavity** | | | | | |
| HR+BC | 3 | 0.6 | 1 | 0.7 | ***<0.001*** |
| HER2+BC | 11 | 2.1 | 2 | 1.5 | ***0.040*** |
| TN-BC | 2 | 0.4 | 0 | 0 | 0.435 |
| **Neurosurgical resection + radiation of resection cavity + focal radiotherapy** | | | | | |
| HR+BC | 0 | 0 | 0 | 0 | ***<0.001*** |
| HER2+BC | 3 | 0.6 | 0 | 0 | ***0.040*** |
| TN-BC | 2 | 0.4 | 0 | 0 | 0.435 |
| **Systemic treatment** | | | | | |
| HR+BC | 0 | 0 | 1 | 0.7 | ***<0.001*** |
| HER2+BC | 5 | 1.0 | 7 | 5.1 | ***0.040*** |
| TN-BC | 0 | 0 | 0 | 0 | 0.435 |
| **BSC** | | | | | |
| HR+BC | 2 | 0.4 | 0 | 0 | ***<0.001*** |
| HER2+BC | 2 | 0.4 | 2 | 1.5 | ***0.040*** |
| TN-BC | 8 | 1.5 | 5 | 3.7 | 0.435 |
| **Intracranial progression after BM diagnosis** | | | | | |
| **Present** | | | | | |
| HR+BC | 99 | 19.1 | 23 | 4.4 | 0.760 |
| HER2+BC | 65 | 12.6 | 23 | 4.4 | 0.573 |
| TN-BC | 64 | 12.4 | 15 | 2.9 | 0.136 |
| **Absent** | | | | | |
| HR+BC | 114 | 22.1 | 24 | 4.6 | 0.760 |
| HER2+BC | 100 | 19.3 | 42 | 8.1 | 0.573 |
| TN-BC | 75 | 14.5 | 9 | 1.7 | 0.136 |
| **Median time from diagnosis of BM to intracranial progression** (in months) | | | | | |
| HR+BC | 15 (range 4-15) | | 27 (range 6-27) | | 0.254 |
| HER2+BC | 18 (range 7-19) | | 31 (range 8-31) | | 0.902 |
| TN-BC | 9 (range 3-9) | | 14 (range 7-15) | | 0.947 |
| **Extracranial progression after BM diagnosis** | | | | | |
| **Present** | | | | | |
| HR+BC | 82 | 15.9 | 25 | 4.8 | 0.085 |
| HER2+BC | 79 | 15.3 | 36 | 7.0 | 0.305 |
| TN-BC | 58 | 11.2 | 13 | 2.5 | 0.658 |
| **Absent** | | | | | |
| HR+BC | 120 | 23.2 | 18 | 3.5 | 0.085 |
| HER2+BC | 86 | 16.6 | 29 | 5.6 | 0.305 |
| TN-BC | 77 | 14.9 | 11 | 2.1 | 0.309 |
| **Median time from diagnosis of BM to extracranial progression** (in months) | | | | | |
| HR+BC | 5 (range 1-14) | | 12 (range 3-12) | | ***0.039*** |
| HER2+BC | 5 (range 3-6) | | 6 (range 4-7) | | 0.148 |
| TN-BC | 4 (range 1-4 ) | | 2 (range 1-7) | | 0.395 |
| **Disease status at the end of life period** (available in 321 patients) | | | | | |
| **Intracranial progression** | | | | | |
| HR+BC | 7 | 1.4 | 3 | 0.6 | 0.795 |
| HER2+BC | 24 | 4.6 | 9 | 1.7 | 0.798 |
| TN-BC | 28 | 5.4 | 6 | 1.2 | 0.339 |
| **Extracranial progression** | | | | | |
| HR+BC | 24 | 4.6 | 7 | 1.4 | 0.795 |
| HER2+BC | 31 | 6.0 | 15 | 2.9 | 0.798 |
| TN-BC | 19 | 3.7 | 1 | 0.2 | 0.339 |
| **Combined progression** | | | | | |
| HR+BC | 72 | 13.9 | 16 | 3.1 | 0.795 |
| HER2+BC | 27 | 5.2 | 8 | 1.5 | 0.798 |
| TN-BC | 19 | 3.7 | 5 | 1.0 | 0.339 |
| **Median OS from diagnosis of BM** (in months) | | | | | |
| HR+BC | 9 (range 1-146) | | 29 (range 1-212) | | ***<0.001*** |
| HER2+BC | 12 (range 1-121) | | 24 (range 1-88) | | ***0.026*** |
| TN-BC | 6 (range 1-91) | | 12 (range 1-64) | | ***0.031*** |

**Abbreviations:** BC: Breast cancer; BM: Brain metastases; BSC: Best supportive care; HR-BC: HER2 (human epidermal growth factor receptor 2)-negative breast cancer; HER2-BC: HER2 overexpressing breast cancer KPS: Karfnofsky Performance Scale; OS: Overall survival; TN-BC: triple-negative breast cancer; WBRT: Whole brain radiation therapy
