# Supplementary material for: Prognostic relevance of the neurological symptom burden in brain metastases from breast cancer
Source: Br J Cancer. 2025 Mar 1;132(8):733–43. doi: 10.1038/s41416-025-02967-w (PMC11997164; doi:10.1038/s41416-025-02967-w)
Supplement: Supplementary file 3 — Supplementary Table 3 [file 41416_2025_2967_MOESM3_ESM.docx]

**Supplementary Table 3:** Characterization of neurological symptoms in symptomatic patients according to BC subtypes *(HR-BC: n=213; HER2-BC: n=165; TN-BC: n= 139)*

| **NEUROLOGICAL SYMPTOMS AT BM DIAGNOSIS IN BC SUBTYPES** | | |  |
| --- | --- | --- | --- |
| **Focal deficits** | **n** | **%** | **p-value** |
| HR+BC | 187 | 87.8 | ***0.005*** |
| HER2+BC | 136 | 82.4 |  |
| TN-BC | 133 | 95.7 |  |
| **Motor disorders** |  |  |  |
| HR+BC | 25 | 11.7 | 0.145 |
| HER2+BC | 14 | 8.5 |  |
| TN-BC | 19 | 13.7 |  |
| **Hemiparesis** |  |  |  |
| HR+BC | 14 | 6.6 | 0.106 |
| HER2+BC | 15 | 9.1 |  |
| TN-BC | 17 | 12.2 |  |
| **Ataxia** |  |  |  |
| HR+BC | 26 | 12.2 | 0.577 |
| HER2+BC | 20 | 12.1 |  |
| TN-BC | 23 | 16.5 |  |
| **Cranial nerve palsy** |  |  |  |
| HR+BC | 53 | 24.9 | 0.490 |
| HER2+BC | 42 | 25.5 |  |
| TN-BC | 26 | 18.7 |  |
| **Hypesthesia** |  |  |  |
| HR+BC | 24 | 11.3 | 0.172 |
| HER2+BC | 16 | 9.7 |  |
| TN-BC | 21 | 15.2 |  |
| **Aphasia** |  |  |  |
| HR+BC | 23 | 10.8 | 0.647 |
| HER2+BC | 23 | 13.9 |  |
| TN-BC | 21 | 15.1 |  |
| **Vertigo** |  |  |  |
| HR+BC | 101 | 47.4 | 0.057 |
| HER2+BC | 81 | 49.1 |  |
| TN-BC | 61 | 43.9 |  |
| **Signs of increased intracranial pressure** |  |  |  |
| HR+BC | 90 | 42.3 | 0.127 |
| HER2+BC | 90 | 54.5 |  |
| TN-BC | 68 | 48.9 |  |
| **Headache** |  |  |  |
| HR+BC | 69 | 32.4 | 0.099 |
| HER2+BC | 68 | 41.2 |  |
| TN-BC | 56 | 40.3 |  |
| **Nausea & Emesis** |  |  |  |
| HR+BC | 25 | 11.7 | 0.119 |
| HER2+BC | 23 | 13.9 |  |
| TN-BC | 15 | 10.8 |  |
| **Epileptic seizures** |  |  |  |
| HR+BC | 36 | 16.9 | 0.747 |
| HER2+BC | 26 | 15.8 |  |
| TN-BC | 18 | 12.9 |  |
| **Focal seizures** |  |  |  |
| HR+BC | 17 | 7.9 | 0.418 |
| HER2+BC | 8 | 4.8 |  |
| TN-BC | 6 | 4.3 |  |
| **Generalized seizures** |  |  |  |
| HR+BC | 16 | 7.5 | 0.440 |
| HER2+BC | 15 | 9.1 |  |
| TN-BC | 8 | 5.7 |  |
| **Focal & generalized seizures** |  |  |  |
| HR+BC | 2 | 0.9 | 0.482 |
| HER2+BC | 3 | 1.8 |  |
| TN-BC | 1 | 0.7 |  |
| **Neuropsychological symptoms** |  |  |  |
| HR+BC | 82 | 38.5 | 0.426 |
| HER2+BC | 55 | 33.3 |  |
| TN-BC | 47 | 33.8 |  |
| **Organic brain disorder** |  |  |  |
| HR+BC | 14 | 6.6 | 0.334 |
| HER2+BC | 7 | 4.2 |  |
| TN-BC | 11 | 7.9 |  |
| **Cognitive dysfunction/impairment** |  |  |  |
| HR+BC | 68 | 17.1 | 0.682 |
| HER2+BC | 48 | 5.6 |  |
| TN-BC | 36 | 25.9 |  |

**Abbreviations:** BC: Breast cancer; BM: Brain metastases; HR-BC: HER2 (human epidermal growth factor receptor 2)-negative breast cancer; HER2-BC: HER2 overexpressing breast cancer; TN-BC: triple-negative breast cancer
