# Supplementary material for: Prognostic relevance of the neurological symptom burden in brain metastases from breast cancer
Source: Br J Cancer. 2025 Mar 1;132(8):733–43. doi: 10.1038/s41416-025-02967-w (PMC11997164; doi:10.1038/s41416-025-02967-w)
Supplement: Supplementary file 4 — Supplementary Table 4 [file 41416_2025_2967_MOESM4_ESM.docx]

**Supplementary Table 4** Treatment strategies and median OS according to symptomatic status and year of BM diagnosis.

|  | **Symptomatic**  **Patients** | | **Asymptomatic**  **Patients** | | | |
| --- | --- | --- | --- | --- | --- | --- |
|  | n=573 | % of n | n=143 | % of n |  |  |
| **Initial treatment strategy after BM diagnosis according to year of diagnosis** | | | | | ***p-value*** | |
| **1992-1999 (n=21)** | | | | | | |
| Focal radiotherapy | 2 | 0.34 | 0 | 0 | | ***<0.001*** |
| WBRT | 1 | 0.2 | 0 | 0 | |  |
| WBRT + focal radiotherapy | 0 | 0 | 0 | 0 | |  |
| Neurosurgical resection | 10 | 1.7 | 0 | 0 | |  |
| Neurosurgical resection + focal radiotherapy | 0 | 0 | 1 | 0.7 | |  |
| Neurosurgical resection + WBRT | 7 | 1.2 | 0 | 0 | |  |
| Neurosurgical resection + WBRT + focal radiotherapy | 0 | 0 | 0 | 0 | |  |
| Neurosurgical resection + radiation of resection cavity | 0 | 0 | 0 | 0 | |  |
| Neurosurgical resection + radiation of resection cavity + focal radiotherapy | 0 | 0 | 0 | 0 | |  |
| Systemic therapy | 0 | 0 | 0 | 0 | |  |
| BSC | 0 | 0 | 0 | 0 | |  |
| **2000-2009 (n=293)** | | | | | | |
| Focal radiotherapy | 89 | 15.5 | 19 | 13.3 | | ***0.005*** |
| WBRT | 68 | 11.9 | 17 | 11.8 | |  |
| WBRT + focal radiotherapy | 38 | 6.6 | 1 | 0.7 | |  |
| Neurosurgical resection | 24 | 4.2 | 6 | 4.2 | |  |
| Neurosurgical resection + focal radiotherapy | 2 | 0.3 | 0 | 0 | |  |
| Neurosurgical resection + WBRT | 10 | 1.7 | 8 | 5.6 | |  |
| Neurosurgical resection + WBRT + focal radiotherapy | 2 | 0.3 | 0 | 0.7 | |  |
| Neurosurgical resection + radiation of resection cavity | 1 | 0.2 | 2 | 1.4 | |  |
| Neurosurgical resection + radiation of resection cavity + focal radiotherapy | 0 | 0 | 0 | 0 | |  |
| Systemic therapy | 2 | 0.3 | 0 | 0 | |  |
| BSC | 2 | 0.3 | 1 | 0.7 | |  |
| **2010-2020 (n=402)** | | | | | | |
| Focal radiotherapy | 78 | 13.6 | 32 | 22.3 | | ***0.053*** |
| WBRT | 119 | 20.8 | 24 | 16.8 | |  |
| WBRT + focal radiotherapy | 19 | 3.3 | 4 | 2.8 | |  |
| Neurosurgical resection | 32 | 5.6 | 10 | 8.0 | |  |
| Neurosurgical resection + focal radiotherapy | 6 | 1.0 | 2 | 1.4 | |  |
| Neurosurgical resection + WBRT | 14 | 2.4 | 7 | 4.9 | |  |
| Neurosurgical resection + WBRT + focal radiotherapy | 1 | 0.2 | 0 | 0 | |  |
| Neurosurgical resection + radiation of resection cavity | 16 | 2.8 | 2 | 1.4 | |  |
| Neurosurgical resection + radiation of resection cavity + focal radiotherapy | 5 | 0.9 | 0 | 0 | |  |
| Systemic therapy | 5 | 0.9 | 8 | 5.6 | |  |
| BSC | 14 | 2.4 | 7 | 4.9 | |  |
| **Median OS from diagnosis of BM** | **median (95%CI)** | | **median (95%CI)** | | | ***p-value*** |
| 1992-1999 (n=21) | 9 (range 1-14) | | 14 (not given) | | | 0.471 |
| 2000-2009 (n=293) | 8 (range 6-10) | | 19 (range 11-27) | | | ***<0.001*** |
| 2010-2020 (n=402) | 9 (range 7-10) | | 27 (range 17-36) | | | ***<0.001*** |

**Abbreviations:** BM: Brain metastases; BSC: Best supportive care; CI: confidence interval; OS: Overall survival; WBRT: Whole brain radiation therapy.
