# Supplementary material for: Prognostic relevance of the neurological symptom burden in brain metastases from breast cancer
Source: Br J Cancer. 2025 Mar 1;132(8):733–43. doi: 10.1038/s41416-025-02967-w (PMC11997164; doi:10.1038/s41416-025-02967-w)
Supplement: Supplementary file 5 — Supplementary Table 5 [file 41416_2025_2967_MOESM5_ESM.docx]

**Supplementary Table 5:** OS from diagnosis of BM according to specific symptoms in symptomatic patients (n=573)

| **Characteristics** | **Symptomatic Patients (n=573 )** | |
| --- | --- | --- |
|  |  |  |
|  | **OS (median)**  **in months** | **p-value** |
| Focal deficits |  | *0.923* |
| Present | 9 |  |
| Absent | 9 |  |
| Motor disorders |  | 0.307 |
| Present | 9 |  |
| Absent | 10 |  |
| Hemiparesis |  | *0.646* |
| Present | 9 |  |
| Absent | 10 |  |
| Hypesthesia |  | *0.829* |
| Present | 8 |  |
| Absent | 7 |  |
| Aphasia |  | *0.429* |
| Present | 7 |  |
| Absent | 10 |  |
| Cranial nerve palsy |  | *0.816* |
| Present | 6 |  |
| Absent | 8 |  |
| Ataxia |  | *0.976* |
| Present | 8 |  |
| Absent | 9 |  |
| Vertigo |  | *0.944* |
| Present | 9 |  |
| Absent | 10 |  |
| Signs of increased intracranial pressure |  | *0.140* |
| Present | 9 |  |
| Absent | 10 |  |
| Headache |  | *0.472* |
| Present | 9 |  |
| Absent | 9 |  |
| Nausea & Emesis |  | *0.555* |
| Present | 9 |  |
| Absent | 8 |  |
| Epileptic seizures |  | *0.514* |
| Present | 10 |  |
| Absent | 9 |  |
| Focal seizures |  | *0.369* |
| Present | 9 |  |
| Absent | 10 |  |
| Generalized seizures |  | *0.221* |
| Present | 9 |  |
| Absent | 9 |  |
| Neuropsychological symptoms |  | *0.041* |
| Present | 4 |  |
| Absent | 8 |  |
| Organic brain disorder |  | *0.303* |
| Present | 7 |  |
| Absent | 10 |  |
| Cognitive dysfunction/impairment |  | ***<0.001*** |
| Present | 6 |  |
| Absent | 12 |  |

Abbreviation: OS: Overall survival
