# Supplementary material for: Prognostic relevance of the neurological symptom burden in brain metastases from breast cancer
Source: Br J Cancer. 2025 Mar 1;132(8):733–43. doi: 10.1038/s41416-025-02967-w (PMC11997164; doi:10.1038/s41416-025-02967-w)
Supplement: Supplementary file 6 — Supplementary Table 6 [file 41416_2025_2967_MOESM6_ESM.docx]

**Supplementary Table 6:**

(A) Multivariate Analysis including neurological symptoms and established prognostic factors;

(B) Internal validation using bootstrapping techniques of neurological symptoms and Breast-GPA

| **MULTIVARIATE ANALYSIS (A)** | | | | **GPA data available in 654 patients** | | | | | | |
| --- | --- | --- | --- | --- | --- | --- | --- | --- | --- | --- |
| **Variable** | | | | **Hazard ratio** | **p-value** | **Confidence interval** | | | | |
| Breast-GPA | | | | 1.4 | ***<0.001*** | 1.3 | | | 1.5 | |
| Neurological symptoms | | | | 1.6 | ***<0.001*** | 1.4 | | | 1.9 | |
| Treatment approach after BM diagnosis | | | | 1.1 | ***0.04*** | 1.03 | | | 1.2 | |
| Size of BM | | | | 1.0 | 0.326 | 0.9 | | | 1.1 | |
| **BOOTSSTRAPING ANALYSIS (B)** | | | | | | | | | | |
| **Variable** | **Original beta** | **Bias** | **Stand. Error** | | **Mean beta** | | **95% CI** | **HR** | | **95% CI HR** |
| Neurological symptoms | 0.49 | 0.0082 | 0.096 | | 0.50 | | [0.3236,0.689] | 1.65 | | [1.3821,1.9917] |
| Breast-GPA | 0.51 | 0.00043 | 0.076 | | 0.51 | | [0.3625,0.6573] | 1.66 | | [1.4369,1.9295] |

Abbreviations: BM: Brain metastases; Breast-GPA: Breast cancer-specific Graded Prognostic Assessment
