# Supplementary material for: Prognostic relevance of the neurological symptom burden in brain metastases from breast cancer
Source: Br J Cancer. 2025 Mar 1;132(8):733–43. doi: 10.1038/s41416-025-02967-w (PMC11997164; doi:10.1038/s41416-025-02967-w)
Supplement: Supplementary file 7 — Supplementary Table 7 [file 41416_2025_2967_MOESM7_ESM.docx]

**Supplementary Table 7:** Prognostic impact of neurological symptoms in Breast-GPA classes

| **PROGNOSTIC SCORE** | | **GPA data available in 654 patients** | | |
| --- | --- | --- | --- | --- |
|  | **n** | **%** | **OS (median)**  **in months** | **p-value*** |
| **SYMPTOMATIC PATIENTS (n= 519)** | | | | |
| **Breast-GPA classes** |  |  |  |  |
| Class I (score: 3.5-4.0) | 124 | 23.9 | 19 | *0.023* |
| Class II (score: 2.5-3.0) | 213 | 41.0 | 11 | *<0.001* |
| Class III (score: 1.5-2.0) | 155 | 29.9 | 5 | *0.013* |
| Class IV (score: 0.0-1.0) | 27 | 5.2 | 3 | *0.027* |
| **ASYMPTOMATIC PATIENTS (n= 135)** | | | | |
| **Breast-GPA classes** |  |  |  |  |
| Class I (score: 3.5-4.0) | 31 | 23.0 | 28 | *0.023* |
| Class II (score: 2.5-3.0) | 60 | 44.0 | 21 | *<0.001* |
| Class III (score: 1.5-2.0) | 38 | 28.1 | 10 | *0.013* |
| Class IV (score: 0.0-1.0) | 6 | 4.4 | 9 | *0.027* |

Abbreviations: Breast-GPA: Breast cancer-specific Graded Prognostic Assessment, OS: Overall survival
